# Magnetic fields generated by submarine power cables have a negligible effect on the swimming behavior of Atlantic lumpfish (*Cyclopterus lumpus*) juveniles

**DOI:** 10.7717/peerj.14745

**Published:** 2023-01-23

**Authors:** Caroline M. F. Durif, Daniel Nyqvist, Bastien Taormina, Steven D. Shema, Anne Berit Skiftesvik, Florian Freytet, Howard I. Browman

**Affiliations:** 1 Ecosystem Acoustics Group, Institute of Marine Research, Storebø, Norway; 2 Ingegneria dell’Ambiente, del Territorio e delle Infrastrutture, Politecnico di Torino, Torino, Italy; 3 Institute of Marine Research, Bergen, Norway; 4 Grótti ehf, Reykjavik, Iceland; 5 Unaffiliated, Storebø, Norway

**Keywords:** Anthropogenic MF, Lumpsucker, Swimming speed, Subsea cables, HVDC

## Abstract

Submarine power cables carry electricity over long distances. Their geographic distribution, number, and areal coverage are increasing rapidly with the development of, for example, offshore wind facilities. The flow of current passing through these cables creates a magnetic field (MF) that can potentially affect marine organisms, particularly those that are magnetosensitive. The lumpfish (*Cyclopterus lumpus*) is a migratory species that is widely distributed in the North Atlantic Ocean and Barents Sea. It migrates between coastal spawning grounds and pelagic offshore feeding areas. We tested whether lumpfish respond to MFs of the same intensity as those emitted by high voltage direct current (HVDC) submarine power cables. Laboratory experiments were conducted by placing juvenile lumpfish in an artificial MF gradient generated by a Helmholtz coil system. The intensity of the artificial MF used (230 µT) corresponded to the field at 1 m from a high-power submarine cable. The fish were filmed for 30 min with the coil either on or off. Swimming speeds, and presence in the different parts of a raceway, were extracted from the videos and analyzed. Juvenile lumpfish activity, defined as the time that the fish spent swimming relative to stationary pauses (attached to the substrate), and the distance travelled, were unaffected by exposure to the artificial MF. The swimming speed of juvenile lumpfish was reduced (by 16%) when the coil was on indicating that the fish could either sense the MF or the induced electric field created by the movement of the fish through the magnetic field. However, it seems unlikely that a 16% decrease in swimming speed occurring within 1 m of HVDC cables would significantly affect Atlantic lumpfish migration or homing.

## Introduction

Marine renewable energy sources such as wind and waves can provide alternatives to fossil fuels. Power generated by offshore energy production facilities is carried over long distances through submarine power cables. Submarine power cables are also used to connect autonomous grids and supply power to islands, marine platforms, or subsea observatories (Taormina et al., 2018). In 2015, the total length of cables (communication and power) laid down on the seabed reached 1,000,000 km, 8,000 km of which represented high voltage direct current (HVDC) power cables (Ardelean & Minnebo, 2015). HVDC cables can be buried or lie on top of the bottom. HVDC power cables produce an electric field (either DC, a static current or AC, time-varying), that is typically retained inside the cable by shielding, and a MF produced by the current passing through them. These MFs are detectable at distances of approximately 10 m from the cable, and may thus affect organisms present in close proximity (Brewer, 1979; Bochert & Zettler, 2004; Normandeau, Tricas & Gill, 2011; Hutchison et al., 2020). The MF generated by HVDC cables may reach 3,200 µT, although after travelling through only 1 m of water it decreases by 10-fold (Taormina et al., 2018). With the increasing number of offshore wind parks inside which turbines are connected by vertical and horizontal cabling, magnetic disturbances will affect organisms in the water column as well as those on or near the bottom (Soares-Ramos et al., 2020).

Many animal species are magnetosensitive and, as a result, may be particularly susceptible to artificial MF (Formicki, Korzelecka-Orkisz & Tański, 2019). Delayed hatching, increased heart rate, increased rate of yolk sac absorption, increased oxygen uptake and changes on the distribution of melanophores have been observed in fish exposed to MFs (Formicki & Perkowski, 1998; Formicki & Winnicki, 1998; Skauli, Reitan & Walther, 2000; Fey et al., 2019; Brysiewicz & Formicki, 2019). Changes in movement behavior, often an attraction to higher intensity MFs (Tanski et al., 2005; Scott, Harsanyi & Lyndon, 2018; Formicki et al., 2004b) or increased motor activity during a geomagnetic storm (Muraveiko, Stepanyuk & Zenzerov, 2013), have also been reported. Animals that use the Earth’s MF to orient or navigate are assumed to be more vulnerable to anthropogenic MF (*e.g*., Phillips, 1996; Boles & Lohmann, 2003; Cresci et al., 2019; Lohmann & Lohmann, 2019), in particular long-distance migrants such as marine mammals, sea turtles and fishes (*e.g*., Walker et al., 1992; Lohmann, Luschi & Hays, 2008; Willis et al., 2009; Vanselow et al., 2018, Durif et al., 2022).

The Atlantic lumpfish (*Cyclopterus lumpus*), a migratory species, is widely distributed between North America, Greenland, Iceland, and Norway. It is rarely found south of the English Channel but is common in the North Sea and the Baltic. Reproductive migrations take place in the spring between the open ocean and their coastal breeding grounds. Adults spawn in shallow waters (<10 m) and a few months after hatching, juveniles migrate offshore (Daborn & Gregory, 1983; Davenport, 1985). Female lumpfish that have spawned during the previous year tend to return to the same area to spawn (Kennedy & Olafsson, 2019), indicative of homing (*i.e*., returning to where they were spawned). There is also evidence of genetically distinct populations (Pampoulie et al., 2014; Whittaker, Consuegra & de Leaniz, 2018), which is also consistent with homing. When they are in the open ocean, lumpfish move throughout the water column from the surface to as deep as 308 m, displaying both pelagic and demersal behavior (Kennedy et al., 2015).

Given the extensive vertical and horizontal migrations undertaken by lumpfish we tested whether their swimming dynamics were affected by MFs emitted by submarine power cables. To do this, we placed juvenile lumpfish in an artificial MF gradient simulating that generated by HVDC cables and tracked their swimming behavior when the coil system was either turned on or off.

## Materials and Methods

### Specimen origin and maintenance

Lumpfish eggs were obtained from Skærneset fisk AS and reared at the Institute of Marine Research’s (IMR) Austevoll Research Station in Austevoll, Norway. They hatched on May 3rd, 2018. The fish were 7 months-old when the experiments were carried out (mean weight (±sd) = 24 ± 4 g, mean size = 8 ± 1 cm). The Austevoll Research Station has a permit to operate as a Research Animal facility for fish (all developmental stages), under Code 93 from the national Institutional Animal Care and Use Committee (IACUC), NARA. All experimental protocols and procedures were performed in accordance with approved guidelines.

### Helmholtz coils

**Figure 1:**
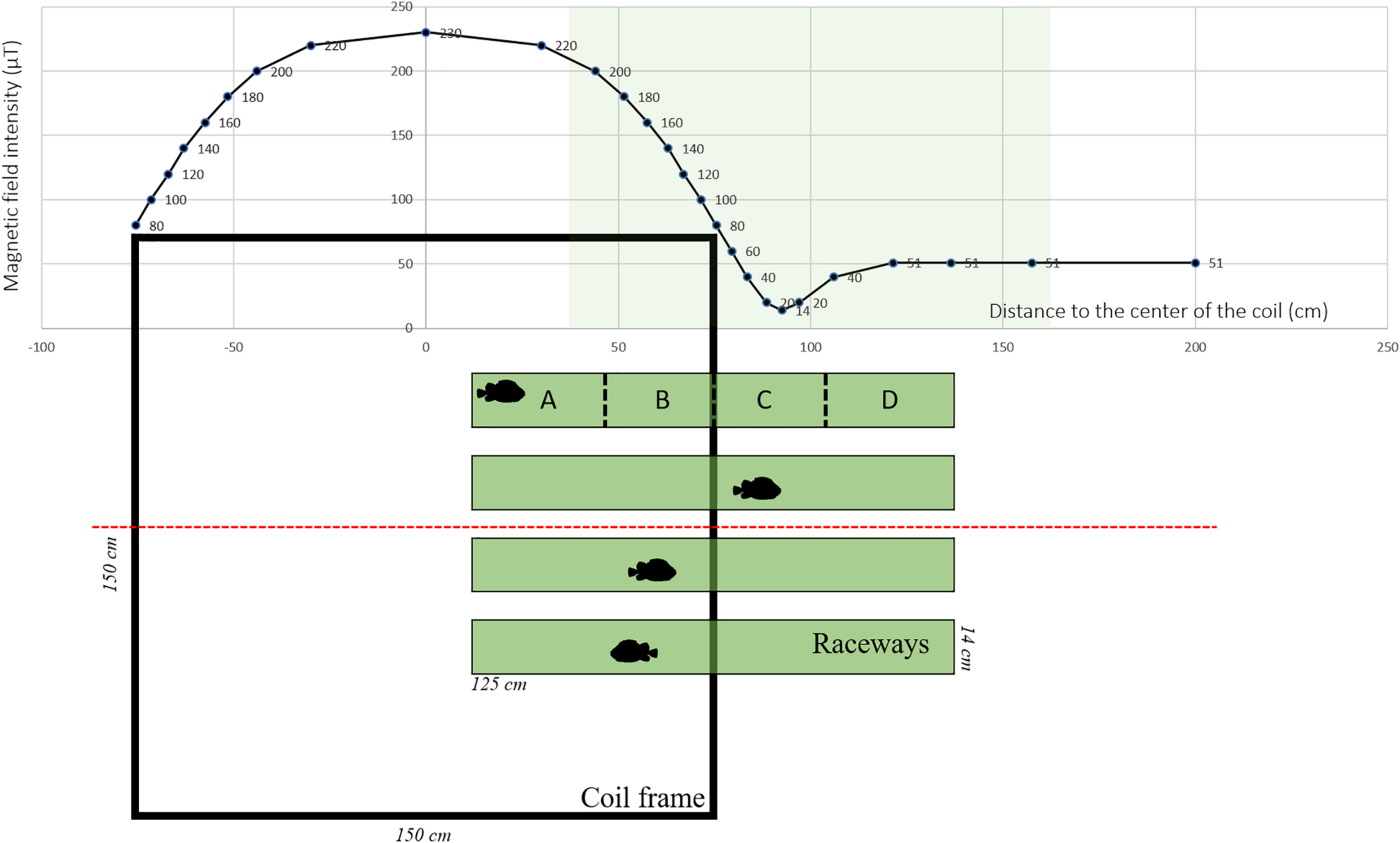
Experimental set-up used to test the effect of electromagnetic fields on the behavior of Atlantic lumpfish (*Cyclopterus lumpus*). Four identical raceways were placed halfway inside a coil system (viewed from the top) which delivered electromagnetic fields of the amplitude of those generated by submarine power cables. For descriptive and analytical purposes, the raceways were divided into four quadrants denoted A, B, C, D. Magnetic field values correspond to measurements made along the red dashed line.

The MF was generated using Helmholtz coils designed by MAPPEM Geophysics© (http://www.mappem-geophysics.com/). The system is described in detail by Taormina et al. (2020). Each of the two coils consisted of 600 m of wire (composed of copper with a 2.5 mm^2^ section) wrapped around a 1.5 m × 1.5 m square wooden frame. The coils were parallel to the floor, therefore only the vertical component of the ambient MF was modified. The coil system (1.5 m × 1.5 m × 1.0 m) produced static (*i.e*., DC) MFs with intensities reaching 230 µT, which is comparable to those produced close (1 m) to HVDC submarine cables (Taormina et al., 2020). Based on the formula: B (µT) = 0.2 * I/d, where I is the current flowing into the wire and d the distance to the wire, 200 µT corresponds to the intensity found at a distance of 1 m from a 1,000 A DC power cable. Each of the two coils was powered by a 15 V electrical current generated by a BK Precision DC power supply (model BK-1745A). The coils created an area of homogeneous MF in the center, and an area of decreasing MF along a gradient towards the periphery (Fig. 1). The natural ambient geoMF field outside of the Austevoll Research Station has an intensity of 51 µT. The MF generated by the coil was measured with a smartphone (PhysicsToolbox magnetometer) along the central axis in the middle of the coil system (Fig. 1).

### Testing protocol

Four white rectangular plastic raceways (125 cm × 14 cm × 7 cm) were placed halfway inside the coil (*i.e*., one end was inside the coil and the other end outside), creating an intensity gradient (Fig. 1). Within the raceway, four different quadrants were defined *a posteriori* denoted A, B, C, and D, corresponding to progressively decreasing intensities with D being the lowest (Fig. 1). As a control, the coil was turned off, so there was no MF gradient inside the raceway. Each raceway was filled with 3 cm of seawater. Water temperature was between 7.5 °C and 8.1 °C and it was replaced between each trial.

On the morning of the trial (28 November 2018), approximately 100 lumpfish were moved from the rearing facility (1 km displacement) to a 100-l holding tank with an aeration system, located in the same room as the coil. The water temperature was the same as in the raceways.

Before each trial, the overhead lights were turned off—to avoid visual cues from the observer manipulating the fish—and four fish were placed inside plastic cylinders (release devices) placed beforehand in the middle of each raceway. After a few minutes the fish were released from the four cylinders by simultaneously lifting them. The observer then left the room and turned on the lights. The fish were filmed for 30 min using a GoPro camera. At the end of each trial, the fish were weighed and measured. Four new fish were collected from the holding tank for each new trial. Treatment (*n* = 6) and control trials (*n* = 6) were alternated and a total of 24 fish were tested per treatment (48 fish for the whole experiment). The coil created a shade on the part of the raceways that was placed inside the coil, but the shading was identical between control and treatment. Therefore, the only external cue that changed was the magnetic field. Further analyses did not reveal any preferences regarding the light gradient.

### Image analysis

Videos were analyzed using ImageJ (Schneider, Rasband & Eliceiri, 2012); the fish were tracked using the “Manual Tracking” plugin. Positions were recorded every 0.5 s over the course of the full recording, that is, after the release device was lifted and the overhead light was turned on. Distance and speed were measured in cm/s.

We extracted the following variables for the analyses: (1) the time spent in each quadrant in min. (A, B, C, D); (2) instantaneous swimming speed in cm/s (over a 0.5 s interval); (3) the total distance travelled in cm/s; and (4) the activity ratio calculated as the amount of time moving divided by the total duration of the trial.

### Data analysis

The body length of the fish was compared between treatments using a t-test. The relationship between the time spent in each quadrant (A, B, C, or D) and treatment (coil on or off) was compared using Cramer’s V test (Cramer, 1946). Cramer’s V is a measure of association between nominal variables giving a value between 0 and +1 and is based on Pearson’s chi-squared statistic.

Differences in the activity ratio of each fish between each treatment and quadrant were tested using a two-way ANOVA followed by a Tukey test. Total distance travelled by each fish was compared between treatment using a t-test. Instantaneous swimming speeds were tested using a Linear Mixed Effect model (LME, package lme4, (Bates et al., 2015)) to account for possible individual differences between the fish. The analyses included treatment as a fixed effect and individual fish as a random intercept. We used a likelihood ratio to test the significance of the treatment effect in the LME. Only speeds >0.5 cm/s were retained to remove immobile fish. Swimming speeds near the edges, quadrants A and D, were removed because of the edge effect. In other words, fish automatically slowed down as they approached both ends of the raceway. A Pearson correlation coefficient between mean velocity per fish and trial numbers was calculated to evaluate whether the time spent in the holding tank before the trial had an effect on the fish. Assumptions of normality and homogeneity, when relevant, were confirmed by visualizing Q-Q plots and histograms of the residuals, residual-fit plots and residual lag plots. All data analysis was carried out with R (R Core Team, 2021).

## Results

Mean body length of lumpfish used in the trials varied between 6.5 and 9.1 cm, but the means (mean ± standard deviation: coil on = 7.98 ± 0.55 and coil off = 8.03 ± 5.54 cm) were not significantly different between treatments (t-test, df = 46, *p* = 0.75).

**Figure 2:**
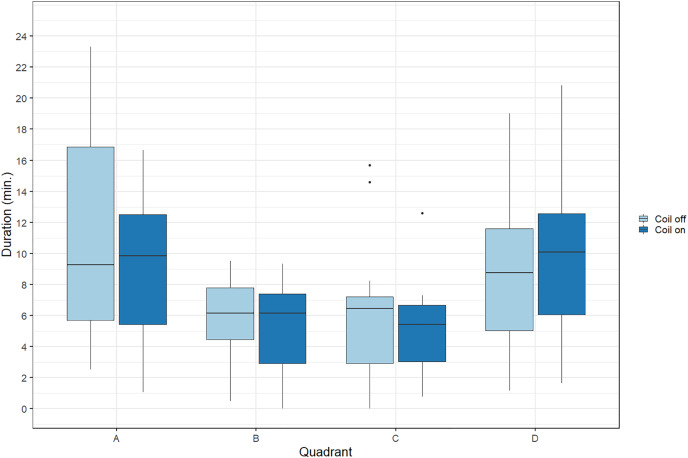
Time spent by Atlantic lumpfish (*Cyclopterus lumpus*) in the four different quadrants of the test raceways. Data are represented as boxplots (median, 25^th^ and 75^th^ percentiles and potential outliers). Quadrants A and B were located in the coil system, resulting in decreasing magnetic intensities from A to D, when the coil was turned on.

**Figure 3:**
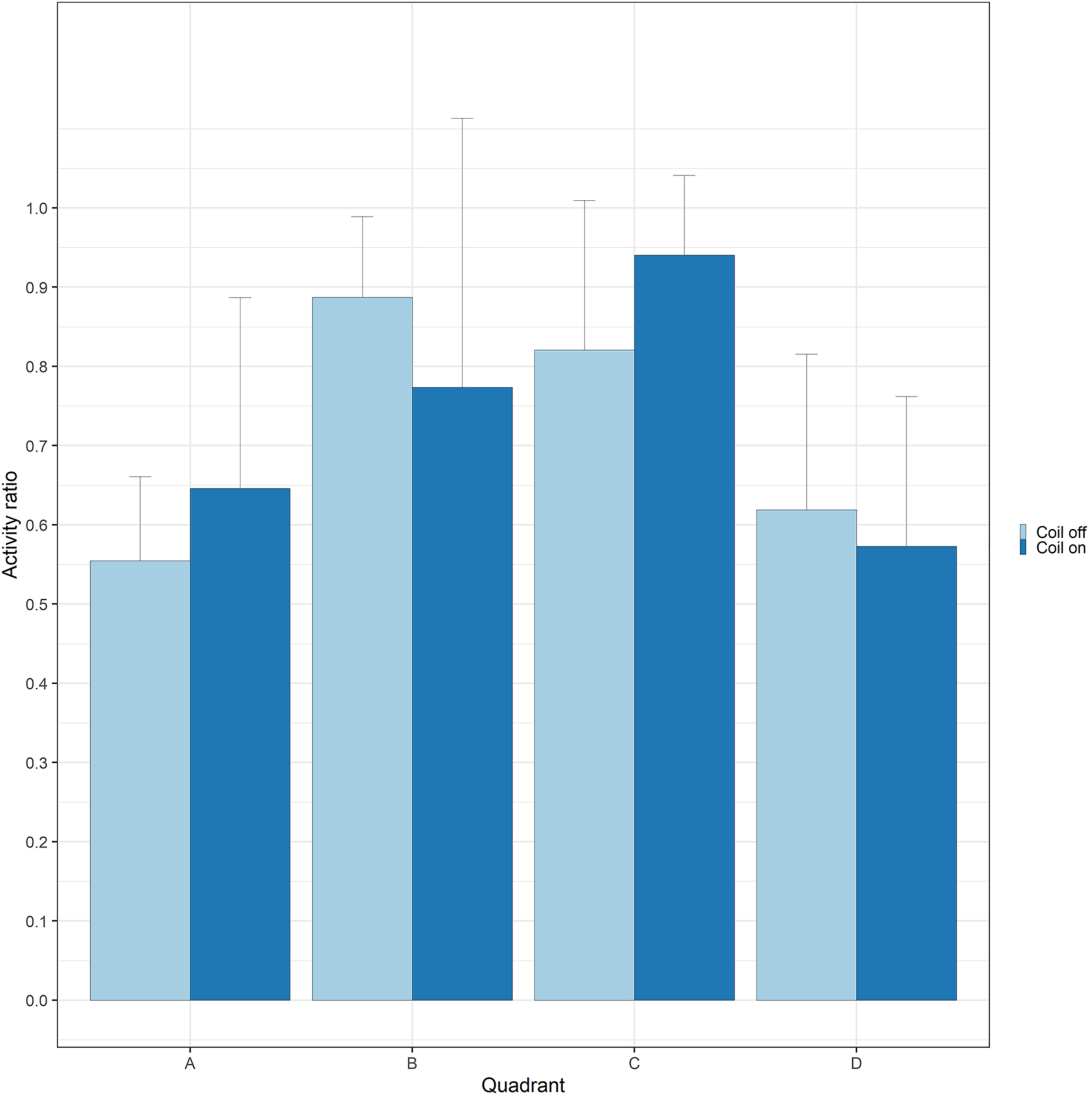
Activity of lumpfish exposed to a gradient of electromagnetic field. Activity (time moving divided by total duration of the trial) of Atlantic lumpfish (*Cyclopterus lumpus*) exposed to a gradient of electromagnetic field (quadrant A: highest, quadrant D is lowest) created by a coil system that was either on or off. Bars represent standard deviations.

**Figure 4:**
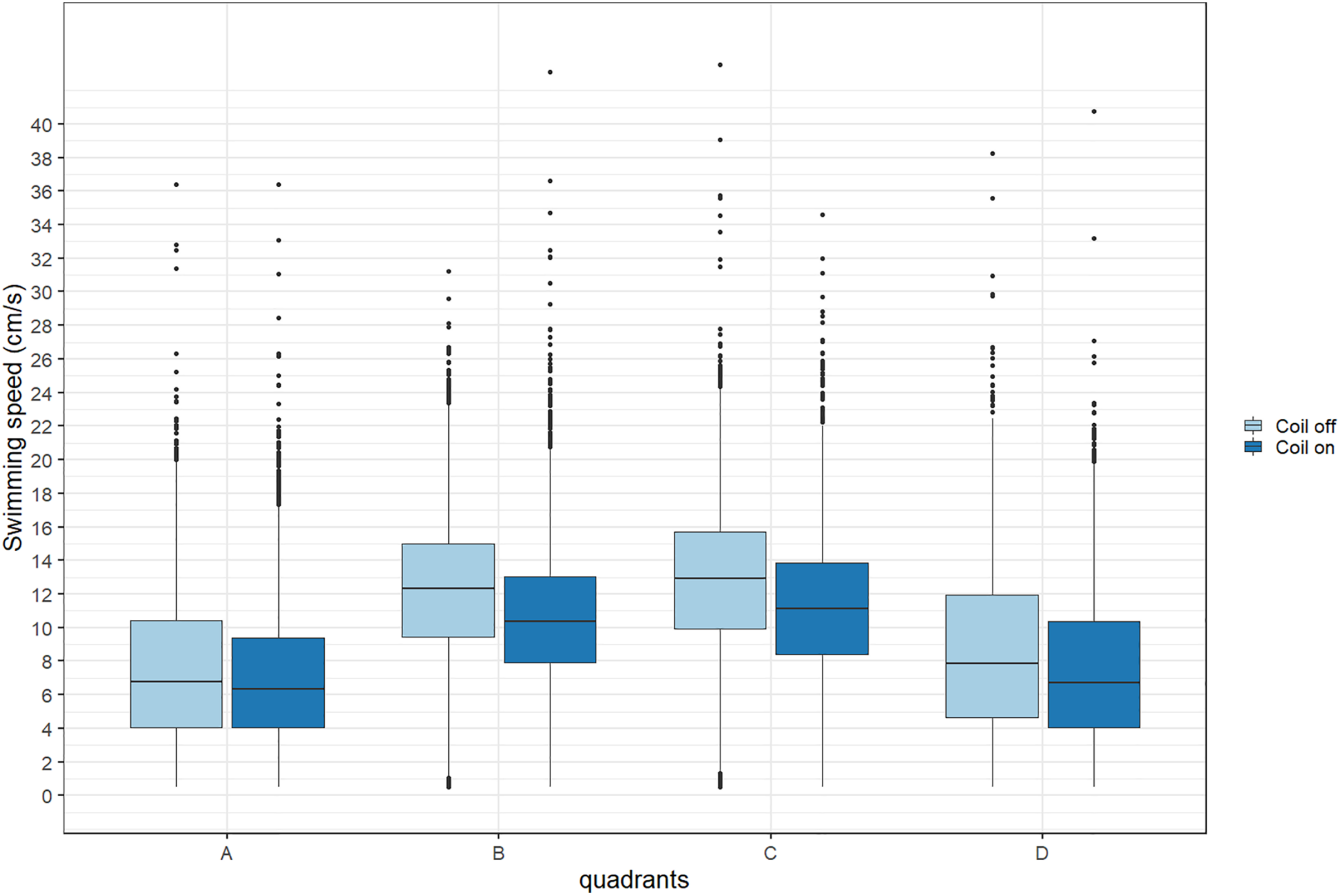
Average swimming speed (cm/s) of Atlantic lumpfish (*Cyclopterus lumpus*) exposed to an electromagnetic field gradient. Quadrant A corresponds to the highest magnetic field, quadrant D to the lowest. The magnetic field was created by a coil system that was either on or off. Boxplots represent the median, the 25^th^ and 75^th^ percentiles and potential outliers.

During trials, lumpfish swam back and forth between both ends of the raceways. They spent more time at the extremities (quadrants A and D) compared to the center (Fig. 2). Every individual explored the full length of the raceways. There was no significant effect of the MF on their presence in the different quadrants (Cramer’s V = 0.0143; 0 indicates no association and one indicates a strong association between both variables, here treatment and quadrant). The activity ratio was significantly higher in the middle of the raceways (quadrant C) compared to the edges (quadrants A and D) (ANOVA, *p* = 0.00849), but was not different between treatments (ANOVA, *p* = 0.853) (Fig. 3). There was no significant treatment effect on the total distance travelled by individual fish (t-test, df = 46, *p* = 0.45). However, the fish reduced their speed when the coil was turned on (χ^2^(1) = 4.1176, *p* = 0.042), by approximately 16%: LME: intercept = 12.2 cm/s (CI [10.9–13.5]), treatment effect estimate = −1.9 cm/s (CI [−3.7 to −0.07]). This decrease was visible in all quadrants but was more pronounced in the middle of the raceways, due to the edge effect (Fig. 4). This decrease in speed was not due to the trial sequence since the treatments were alternated, and there was no significant relationship between swimming speed and trial number (r = 0.11; *p* = 0.44).

## Discussion

The activity and distance travelled by juvenile lumpfish were unaffected by exposure to an artificial DC MF, nor were they attracted to/repulsed by an artificial MF gradient. However, the swimming speed of juvenile lumpfish was 16% lower in the presence of an artificial MF. The intensity of the artificial MF used (up to 220 µT) in our experiments corresponded to a field at a distance of 1 m from a high-power submarine cable, so this small effect on swimming speed would be localized nearby a cable.

The observed MF-related decrease in swimming speed of lumpfish was smaller (this study: 16%) than that observed in Atlantic haddock larvae (*Melanogrammus aeglefinus*) (60%) exposed to slightly lower intensities (Cresci et al., 2022a; 150 µT, this study: ~200 µT). European eel (*Anguilla anguilla*) also reduced their swimming speed in the vicinity of a submarine power cable (Westerberg & Lagenfelt, 2008). Observations were made *in situ* as eel were tracked while passing over a cable in the Baltic Sea. This cable delivered 5 µT at 60 m. The speed was proportional to the electric current, although the correlation was weak (Westerberg & Lagenfelt, 2008). Swimming speed of little skate (*Leucoraja erinacea*) was reduced by 29% when exposed to varying MFs, between ‘low’ (mean 48.7 µT) and ‘high’ (mean 60.1 µT), representing a difference of approximately +/− 4 to 10 µT from the Earth’s MF (Hutchison et al., 2020). On the other hand, MFs, up to 150 µT above the Earth’s MF, did not affect the speed of movement of lobster (*Homarus americanus* and *H. Gammarus*) (Hutchison et al., 2020; Taormina et al., 2020), nor did an alternating 600 µT field affect the swimming behavior and orientation of chum salmon (*Onchorhynchus keta*) (Yano et al., 1997). No effects were observed on the swimming speed or distribution of the lesser sandeel (*Amodytes marinus*) (Cresci et al., 2022b).

More subtle behavioral responses to MF exposure were observed in the little skate (Hutchison et al., 2020). These were interpreted as increased exploration (higher proportion of large turns and increased distance travelled), possibly related to search for prey (Hutchison et al., 2020). American lobster tested in the same enclosure system, showed differences in their spatial distribution and swimming behavior when exposed to MF, but for this species no link was established between the intensity of the field and the behavioral effect (Hutchison et al., 2020).

None of the other behavioral variables that we measured (activity, spatial distribution or distance travelled) were different when the fish were exposed to the MF. The lumpfish were neither attracted nor repulsed from the higher magnetic intensities. Trout (*Salmo trutta*) alevins were attracted to magnets generating fields of up to 4,200 µT (Formicki et al., 2004a). Fyke nets rigged with magnets caught more fish (cyprinids and percids) than control nets (Formicki et al., 2004b). Spinycheek crayfish (*Orconectes limosus*) preferred shelters fitted with magnets generating fields between 200 and 800 µT (Tanski et al., 2005) and brown crab (*Cancer pagurus*) were attracted to a magnetic source of 2,800 µT (Scott, Harsanyi & Lyndon, 2018). The higher intensities generated in these experiments may explain the differences in the response of these species compared to our observations on lumpfish.

The effects of anthropogenic MF on adult fishes appear to be subtle, at least when other sensory cues are available. Attempts to use electroMF to guide eel, or as barriers to avoid turbine entrainment at hydroelectric facilities, have been unsuccessful (Pratt et al., 2021). Even at very high intensities (max field = 3,400 µT) electromagnetic fields do not modify the swimming behavior of fish sufficiently for them to avoid turbine entrainment (Electric Power Research Institute, 2016). The large magnetic anomalies produced by either bridges or a high voltage DC power cable in the San Francisco (California, USA) estuary did not affect the passage of migratory Chinook salmon smolts (*Oncorhynchus tshawytscha*) nor of upstream migrating green sturgeon (*Acipenser medirostris*) (Klimley, Wyman & Kavet, 2017).

The ecological impact of the small reduction in swimming speed observed in lumpfish is difficult to assess but would probably be limited. However, it implies that lumpfish sensed the MF. Since lumpfish do not use electroreception, it is unlikely that they sensed the induced electric current produced by their movement through the MF. While the mechanism or driver for the observed reduction in speed of lumpfish is unknown, it could indicate a hesitance in response to the new sensory input. Another explanation could be that the MFs affect the cardiac muscle (Formicki, Korzelecka-Orkisz & Tański, 2019). In fish larvae (*Coreganus lavaretus, Atherina boyeri, Salmo trutta, Cyprinus caprio*), high intensity MF causes variations in heart rate, often by increasing it but, depending on the species, also resulting in more chaotic contractions (Winnicki et al., 2004; Li et al., 2014; Formicki, Korzelecka-Orkisz & Tański, 2019). If lumpfish are indeed magnetosensitive further research is needed to determine how exposure to magnetic anomalies may disrupt their orientation system.

## Conclusions

We tested whether the behavior of Atlantic lumpfish was affected by electroMF of the same intensity as the ones emitted by submarine power cables. The only observed significant change in behavior was a decrease in swimming speed by approximately 16% on average. Whether the reduction in swimming speed would be transient or prolonged is still an open question. It seems unlikely that this decrease in swimming speed, which would occur within 1 m of HVDC cables, would significantly affect lumpfish migration. However, the present study suggests magnetosensitivity in lumpfish which would be consistent with the putative homing behavior. Further experiments should investigate their potential magnetic sense and how anthropogenic MF may affect orientation that is based on perceiving the earth’s geoMF. Disruption of their orientation capabilities would probably have more impact on their survival than the observed decrease in swimming speed.

Whatever the consequence for the species, it might be more important in the context of the increasing development of offshore wind turbines. Effects on offshore-inshore migrants might be accumulated as the fish cross over several cables and travel through wind parks (Normandeau, Tricas & Gill, 2011).

## Supplemental Information

10.7717/peerj.14745/supp-1 Supplemental Information 1 Swimming behavior of lumpfish exposed to a magnetic field. ‘Treatment’ corresponds to the trials when the coil was on, ‘control’ when the coil was off. Distance is in pixels and velocity in pixels/s.

## Additional Information and Declarations

### Data Availability

The following information was supplied regarding data availability: The raw data from the digitalized tracks are available as a Supplemental File.
